# Early Antifungal Treatment in Immunocompromised Patients, Including Hematological and Critically Ill Patients

**DOI:** 10.3390/jof12010059

**Published:** 2026-01-13

**Authors:** Galina Klyasova, Galina Solopova, Jehad Abdalla, Marina Popova, Muhlis Cem Ar, Murat Sungur, Riad El Fakih, Reem S. Almaghrabi, Murat Akova

**Affiliations:** 1 Department of Microbiology and Antimicrobial Therapy, National Medical Research Center for Hematology, 125167 Moscow, Russia; klyasova.g@blood.ru; 2 Infection Control Department, Dmitry Rogachev National Medical Research Center of Pediatric Hematology, Oncology and Immunology, 119571 Moscow, Russia; galigen@yahoo.co.uk; 3 Department of Infectious Diseases, Sheikh Khalifa Medical City, Abu Dhabi 51133, United Arab Emirates; jehadabdalla@aol.com; 4 RM Gorbacheva Research Institute of Pediatric Oncology, Hematology and Transplantation, Pavlov University, 197022 St. Petersburg, Russia; marina.popova.spb@gmail.com; 5 Division of Hematology, Department of Internal Medicine, Cerrahpaşa School of Medicine, Istanbul University-Cerrahpaşa, 34098 Istanbul, Turkey; mcemar68@yahoo.com; 6 Division of Intensive Care, Department of Internal Medicine, School of Medicine, Erciyes University, 38280 Kayseri, Turkey; msungur@erciyes.edu.tr; 7 Department of Hematology, King Faisal Specialist Hospital & Research Center, Riyadh 11471, Saudi Arabia; riadfakih@hotmail.com; 8 College of Medicine, Alfaisal University, Riyadh 11471, Saudi Arabia; 9 Section of Transplant Infectious Diseases, Organ Transplant Centre of Excellence, King Faisal Specialist Hospital and Research Centre, Riyadh 11311, Saudi Arabia; dr_reem2002@icloud.com; 10 Infectious Diseases and Clinical Microbiology, Hacettepe University School of Medicine, 06230 Ankara, Turkey

**Keywords:** invasive fungal diseases, antifungal agents, immunocompromised patients, critically ill patients, opportunistic infection, consensus, Delphi survey, empiric antifungal therapy, early antifungal treatment

## Abstract

(1) Background: Invasive fungal diseases (IFDs) represent significant challenges in clinical practice, particularly among immunocompromised individuals, leading to substantial morbidity and mortality. The present document aims to provide evidence-based consensus for the timely initiation of antifungal treatment, focusing on early empiric approaches among immunocompromised patients. (2) Methods: A multidisciplinary expert panel of nine healthcare professionals (HCPs) reviewed the literature, including guidelines and consensus reports (2013–2023; PubMed, Scopus). The panel defined appropriate empiric antifungal approaches for invasive candidiasis, aspergillosis, and mucormycosis among hematological and critically ill patients. Consensus was defined as ≥75% agreement. (3) Results: A total of 47 statements were included. The experts recommend that early targeted antifungal therapy is critical for high-risk patients with suspected IFDs. Empiric therapy may be initiated before definitive diagnosis, considering the local fungal prevalence and the patient’s risk category. Close monitoring is essential, and switching between antifungal classes may be necessary for patients who experience deterioration or side effects. The transition from intravenous to oral therapy depends on the specific infection, the availability of therapeutic drug monitoring, and the patient’s progress. (4) Conclusions: Implementing this targeted, early approach may improve the outcomes of vulnerable patients with IFDs.

## 1. Introduction

Invasive fungal diseases (IFDs) pose significant challenges in clinical practice, particularly among immunocompromised individuals, as they can lead to substantial morbidity and mortality [1,2,3]. *Aspergillus* species, ubiquitous in the environment, can cause a spectrum of infections, ranging from allergic bronchopulmonary aspergillosis to invasive aspergillosis (IA), particularly affecting immunocompromised hosts [4,5]. On the other hand, *Candida* species are commensals of the human microbiota. Still, they can become opportunistic pathogens, causing candidemia and invasive candidiasis (IC), especially in critically ill patients and those with indwelling medical devices [6]. Mucormycosis, caused by fungi of the order *Mucorales*, is characterized by rapid angioinvasive growth leading to tissue necrosis and has emerged as a concerning healthcare-associated infection in immunocompromised individuals, particularly in the context of hematological malignancies and solid organ transplantation [7].

The diagnosis of IFDs is challenging, as gold standard tests (culture and histopathology) often exhibit low sensitivity. Therefore, physicians commonly rely on clinical signs, imaging, and supplementary tests to establish a diagnosis [8,9,10]. Moreover, nonculture tests such as polymerase chain reaction (PCR) and serological tests have the potential to detect patients with IFDs while shortening the time of diagnosis [11].

Serological tests are becoming increasingly essential in the diagnosis of fungal infections, particularly those caused by *Candida* and *Aspergillus* species among patients with neutropenia and hematological malignancies. They enable the detection of antibodies or antigens in the blood or sterile fluids of the host, such as β-(1,3)-d-glucan (BDG), mannan antigen, antimannan antibody, *Candida albicans* germ tube antibody (CAGTA), galactomannan (GM) antigen, and glucuroxylomannan antigen [12].

GM and BDG are widely used serological assays that can assist in diagnosing IFI. For instance, a recent meta-analysis has demonstrated that serum BDG and GM assays exhibit high accuracy in detecting IA in suspected patients, with areas under the curve of 0.83 and 0.90, respectively. The pooled sensitivity was 0.72 and 0.53, and the pooled specificity was 0.82 and 0.94 for both tests. Furthermore, the diagnostic sensitivity was further improved by combining both assays (0.84) [13].

Detection of fungal BDG in the blood has become a trigger for starting antifungal treatment in suspected or probable IC infections before microbiological confirmation. However, Eades and his colleagues have reported variable sensitivity and specificity of different serological tests for detecting IC. The sensitivity and specificity of the BDG assay were 0.86 and 0.80, respectively [14]. Haydour et al. demonstrated the pooled sensitivity and specificity of the BDG assay for ICs of 0.81 and 0.60, respectively [15]. Christner et al. reported lower values of sensitivity and specificity (0.74 and 0.45, respectively) [16]. This variability might be explained by species and population-specific differences [15,17].

Furthermore, cell-free DNA (cfDNA) PCR is a promising novel alternative diagnostic test for the accurate and rapid noninvasive diagnosis of IFDs. Previous studies have demonstrated that cfDNA PCR has superior sensitivity and specificity for detecting IFDs compared with existing non-invasive diagnostic tests. Thus, utilizing cfDNA PCR could further enhance the prompt diagnosis of IFDs and improve patient outcomes [18,19,20].

With an increasing population of immunosuppressed individuals, the risk of IFD rises, leading to more frequent use of antifungal medications for both preventive and therapeutic purposes. Consequently, this trend contributes to higher expenses associated with antifungal drug utilization [21,22,23,24,25].

IFDs are more common in children who have received allogeneic hematopoietic stem-cell transplant (HSCT), lung transplant, those with primary immunodeficiencies, and acute myeloid leukemia (AML). However, the incidence of IFDs in pediatric patients can vary due to treatment protocols, antifungal prophylaxis, inconsistent diagnostic criteria, and challenges in diagnosis [26,27,28]. Like adults, most children with IA are diagnosed with pulmonary disease, which may spread to the central nervous system in up to 15% of cases. Neonates are more likely to experience invasive cutaneous aspergillosis [29,30,31].

There are several guidelines on various IFDs, including but not limited to the European Conference on Infections in Leukemia (ECIL), Infectious Diseases Society of America (IDSA), and the American Society of Transplantation—Infectious Diseases Community of Practice (AST-IDCOP), suggesting various treatment options to manage IFDs [32,33,34,35,36]. For example, empiric treatment involves initiating therapy based on clinical judgment and suspicion of a specific condition without waiting for definitive diagnostic test results. Determining the length of empiric antibacterial therapy (EAT) and antifungal therapy (AFT) for patients with hematological malignancies and fever with neutropenia of unknown cause can be complex. This is particularly important for those at high risk of neutropenia, which lasts more than a week [35].

This consensus document addresses key questions regarding the optimal approach to empiric therapy, focusing on the timing and selection of treatment strategies in various clinical scenarios. Specifically, we aim to explore the appropriateness and implementation of empiric AFT in various healthcare settings, utilizing decision support tools and considering emerging trends in IFD epidemiology. More precisely, we aim to provide evidence-based consensus and practical insights for the timely initiation of AFT, with a focus on early empiric approaches in immunocompromised patients.

## 2. Materials and Methods

**Figure 1:**
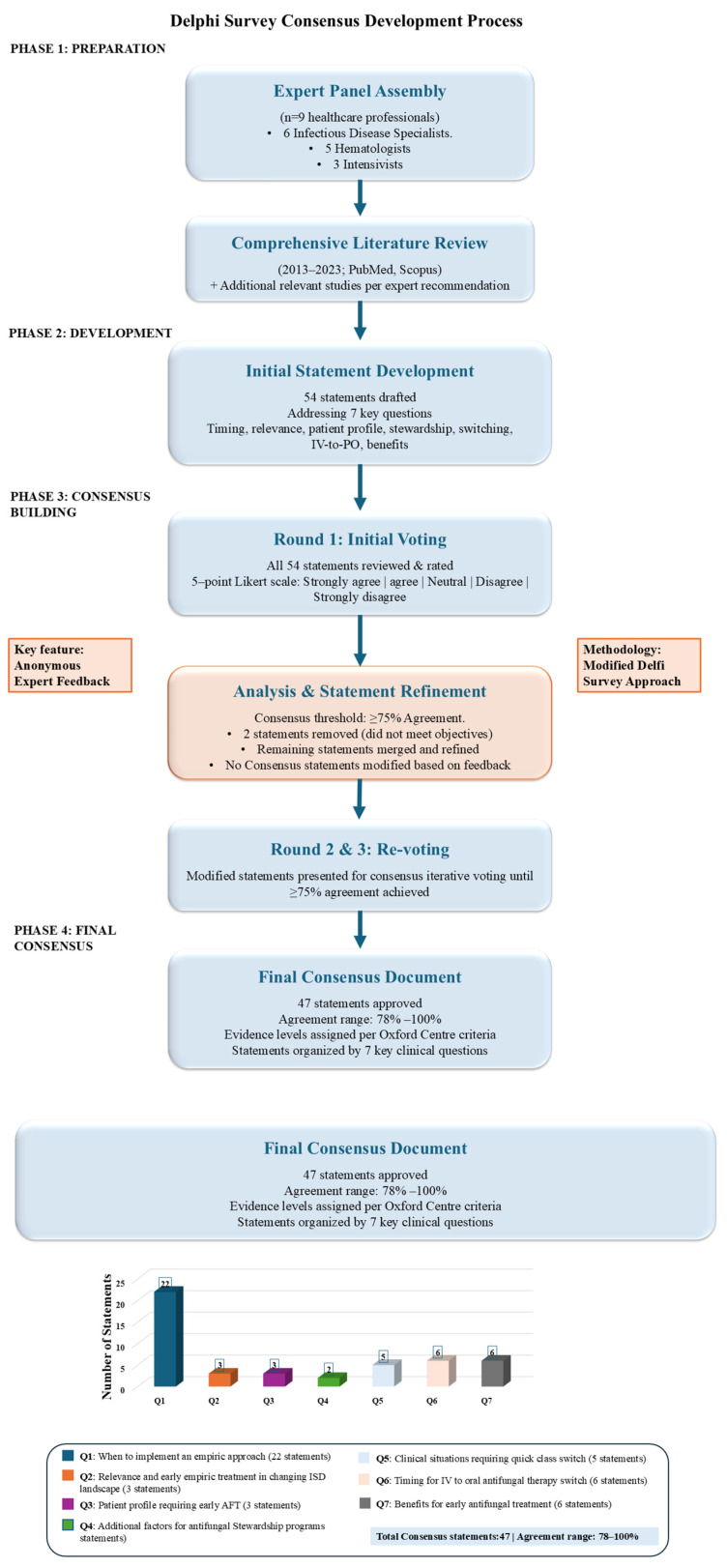
Flowchart of the Delphi Consensus Process.

The Delphi survey was utilized to establish a group consensus on the core list of statements generated by the panel members. In brief, the Delphi survey is a well-described, structured process for building consensus among a group of informed experts, widely used in health research. It involves a series of surveys or “rounds” directed to experts. The Delphi method typically includes the following stages: (1) assembling a panel of experts; (2) assigning forecasting tasks or challenges to the experts; (3) experts submit initial forecasts with justifications, which are then compiled and summarized to provide feedback; and (4) feedback is given to the experts, who review and revise their forecasts based on the feedback. This process may be repeated until a satisfactory level of consensus is reached, and (5) final estimates are created through statistical aggregation of group responses to reach agreement on specific topics. The main features of this method are the participants’ anonymity and the controlled feedback [37]. The systematic process, which ensured iterative feedback and eventual consensus on the final recommendations, is outlined visually in Figure 1.

The present consensus document on early AFT for IFDs reflects the combined expertise of a nominated panel of healthcare professionals from various specialties, including adult and pediatric hematologists, intensivists, and infectious disease specialists. A comprehensive literature search was conducted between 2013 and 2023 across several databases, including PubMed and Scopus, to explore the appropriate use of early AFT for IC, aspergillosis, and invasive mucormycosis (IM) in hematological and critically ill patients. While the initial search adhered to a predefined timeframe, the expert panel recommended including additional relevant studies published outside this window [38,39] for a more complete understanding of IFDs, antifungal options, and especially empiric therapy in children. Search terms included ‘*aspergillus*,’ ‘aspergillosis’, ‘mucormycosis’, ‘*candida*,’ ‘candidiasis’, ‘invasive’, ‘hematology’, ‘antifungal agents’, ‘critically ill’, ‘stem cell transplantation’, and ‘bone marrow transplantation.’

The present consensus employed predefined questions, which were adapted as necessary to ensure clarity and relevance. The seven key predefined questions by the experts were as follows:

1. When is the empiric approach appropriate and should be implemented (what types of centers, diagnostic tools, and clinical situations)?
2. Relevance of early empiric treatment approach in the changing IFD landscape (including epidemiology, between clinical guidelines and clinical practice).
3. What are the clinical situations or patients’ profiles when AF treatment should be prescribed early?
4. Should additional factors (local epidemiology, antifungal prophylaxis) be considered when choosing an antifungal for empiric therapy to form antifungal stewardship programs?
5. What are the clinical situations or patient profiles that require a quick class switch? (Indications for switching between the antifungal classes).
6. What is the best time to switch from intravenous (IV) to per os (PO) antifungal therapy? What are the clinical signs and symptoms that may trigger a switch from IV to PO?
7. What are the benefits of early antifungal treatment in reducing mortality rate, hospital/ICU stay, and time to clinical improvement in different patient populations?

The statements addressing the objectives of the present consensus were formulated. The statements were shared with the panel experts in the first round of the Delphi survey. The experts were asked to rate their level of agreement or disagreement with each statement: strongly agree, agree, neutral, disagree, or strongly disagree. Consensus was defined as ≥75% agreement (strongly agree and agree). The statements that did not achieve 75% agreement were shared with the panel experts in the second and third rounds of the Delphi survey for further discussion, modification, or removal from the survey [40].

The levels of evidence of the consensus statements were graded according to the Oxford Centre for Evidence-Based Medicine’s Levels of Evidence and adapted from Dykewicz et al. [41] (Table A1).

## 3. Results

The consensus was developed by a multidisciplinary panel of nine healthcare professionals (HCPs). Among the panel members were six infectious disease specialists, five hematologists covering both adult and pediatric populations, and three intensivists. Several participants had dual or triple subspecialties.

Initially, 54 statements were drafted and shared with all panel experts. Following the voting, it was determined that two of the statements addressing question 1 had not met the objectives of the present consensus, and the remaining statements were merged with the other statements. Hence, the final number of statements was reduced to 47, successfully approved through three rounds of voting and discussions.

**Q1: When is the empiric approach appropriate and should be implemented (what types of centers, diagnostic tools, and clinical situations)?**

Initially, there were 29 statements addressing this question. After the voting, two statements did not reach a consensus, and the remaining statements were merged. Finally, 22 statements are included.

**a.**  **Hematology patients:**

Statements 1–7 address the abovementioned question among hematological adult patients, whereas statements 8–11 address the same question among hematological children (Table 1). The decision to implement an empiric approach in hematological adult patients depends on various factors, with the severity and duration of neutropenia being critical determinants of risk for IFDs following intensive myelosuppressive chemotherapy [42]. In high-risk patients characterized by significant neutropenia and presenting symptoms, early intervention through empiric therapy is crucial, as waiting for microbiological or histopathological confirmation may delay treatment initiation [35]. There is an agreement of 89% among the experts stating that empiric therapy should be started in settings that lack diagnostic capabilities. However, it is essential to note that empiric therapy should not overshadow the importance of diagnostic efforts, as others recommend through investigations before initiating the empiric treatment [43]. Diagnostic procedures, such as lesion biopsies, chest and sinus radiographs, stains and cultures, and imaging investigations, should be pursued before starting empiric therapy, as reflected by 100% agreement on this statement in Table 1. The diagnostic approach for suspected IA typically involves chest computed tomography (CT) scans, serum and/or bronchoalveolar lavage (BAL) GM antigen detection, and, whenever possible, microscopy examination with fluorescent brighteners that are used as enhancing methods for the detection of fungal elements, such as Calcofluor White (CFW) and Blankophor and histopathological examination of tissues and fluid specimens [44]. We found 100% agreement among experts regarding the diagnostic tools recommended for neutropenic patients with a suspicion of IA (Table 1).

Despite this, fluorescent brighteners that are used as enhancing methods for the detection of fungal elements, such as Calcofluor White and Blankophor, are considered superior to conventional stains such as KOH and Gram staining in terms of sensitivity and specificity, as well as turnaround time; however, they are expensive and not widely available. Hence, conventional stains are still recommended as viable and cost-effective options, as they are inexpensive and routinely used for the immediate diagnosis of fungal infections [45,46].

One challenge associated with empiric AFT is the risk of overtreatment, which can expose patients to unnecessary antifungal toxicities and increase costs. Therefore, a judicious approach, guided by ongoing evaluation, is necessary to determine when to initiate and discontinue AFT in case of suspected breakthrough fungal infection. Empiric therapy using antifungal agents of different classes is recommended until a definitive diagnosis is established. Specifically, lipid formulations of amphotericin B, caspofungin, micafungin, or voriconazole in centers with high IA incidence rates are preferred choices for empiric therapy in hematological patients with neutropenia and fever who have been on broad-spectrum antibiotic therapy for an extended period [44]. The present consensus document records an agreement of 78% for the AFTs mentioned above while considering empiric therapy for hematology patients. Empiric AFT is advised for patients with prolonged neutropenia (>96 h) and fever (>38 °C) receiving parenteral broad-spectrum antibiotics, aiming to reduce the incidence of IA and associated mortality [47]. Conversely, we found an agreement of 89% among experts (Table 1), suggesting that empiric AFT is generally not recommended for patients with anticipated shorter durations of neutropenia unless other clinical findings suggest a potential fungal infection [48].

In pediatric hematological patients, the decision to employ empiric therapy is similar to that in adults, with similar diagnostic approaches utilized, as shown in an 89% agreement for this statement, as presented in Table 1 [44]. The updated guidelines for pediatric patients with fever and neutropenia recommend starting empiric AFT with caspofungin or liposomal amphotericin B for pediatric hematological patients experiencing prolonged (≥96 h) neutropenia that does not respond to broad-spectrum EAT, unless a pre-emptive (diagnostic and test-based approach to receive AFT, at any time of the follow-up, when persistent fever and neutropenia are accompanied by any of the signs or symptoms indicating IFDs) AFT approach is adopted [49]. We found 100% agreement among the experts, suggesting that empiric AFT may be considered between four and seven days of fever without a clear cause and unresponsive to broad-spectrum antibacterial agents, particularly in children with AML, high-risk lymphoblastic leukemia, or relapsed acute leukemia, as well as those with prolonged neutropenia or who have undergone allogeneic HSCT [50]. Pre-emptive administration of AFT demonstrated comparable effectiveness to empiric treatment in children with cancer, fever, and neutropenia, resulting in a notable reduction in the use of antifungal medications [51]. An empiric or pre-emptive strategy for AFT prescription should be preferred based on the availability of diagnostic tools. Caspofungin and liposomal amphotericin B are recommended for empiric treatment in pediatric cases, as indicated by 89% agreement among the experts. In addition, we found an 88% agreement among the experts, suggesting that empiric AFT is typically continued until the condition is resolved in neutropenic children unless there is a suspicion or documentation of invasive fungal disease [50,52,53].

**b.**  **Non-neutropenic ICU patients:**

Statements 12–22 address question 1 among non-neutropenic patients in the ICU.

In critically ill patients admitted to the intensive care unit (ICU), initiating the empiric AFT depends on a multifactorial assessment of clinical presentation, risk factors, and diagnostic findings. Such diagnostic tools as bronchoscopy with fungal cultures and GM testing in BAL fluid play a pivotal role in identifying IA in this population, particularly those with suspected IA [44]. The percentage agreement for this statement is 100%, as illustrated in Table 1. However, there is a 78% agreement, suggesting that serum GM testing is not recommended for non-neutropenic critically ill patients with suspicion of IA [44]. This recommendation was based on the inferior sensitivity and specificity of serum GM testing compared with GM testing in BAL fluid among non-neutropenic ICU patients. Meersseman and his colleagues have previously shown that the sensitivity of GM detection in BAL fluid and the serum was 88% and 42%, respectively [54]. Similarly, Wu et al. have reported the areas under the curve of GM detection in BAL fluid, and the serum were 0.961 and 0.699, respectively, emphasizing that detection of GM in the BAL fluid is more efficient among non-neutropenic ICU patients [55].

Similarly, when IC is suspected, blood, sterile body fluid cultures, and microscopic examination are imperative for diagnosis [56]. The diagnostic approach is further enhanced by integrating conventional culture-based and non-culture-based tests, including serological assays and PCR-based techniques [56].

Conventional methods for detecting fungi in sterile specimens by culture or histopathology usually provide a diagnosis of proven IFI. Despite these techniques being widely available and cost-effective in resource-limited settings, they have limited sensitivity and a slow turnaround time. Non-conventional techniques, including serological and molecular techniques, only provide a diagnosis of probable IFI. These techniques provide rapid, sensitive, and species-specific detection of fungal pathogens or their metabolites. However, serological tests have challenges relevant to the generation of false positives and sensitivity. Furthermore, PCR-based techniques are costly, need specialized equipment, and are limited to a few species in the fungal panels, with challenges related to assay standardization and result interpretation [12].

ICU patients with fever of unknown origin, septic shock with multi-organ failure, multifocal *candida* colonization, prolonged ICU stays, and elevated “Acute Physiology and Chronic Health Evaluation II” (APACHE II) scores may benefit from early antifungal therapy for candidiasis, especially if they present with suspected catheter-related candidemia along with the removal of the catheter [57,58]. Empiric AFT may be considered in critically ill patients such as those with multi-organ failure, septic shock, prolonged ICU stays, high APACHE II scores, *candida* colonization (especially multifocal), and no identifiable cause of fever. The therapy should be based on a clinical assessment of risk factors, supported by one or more surrogate markers (Beta-D-glucan, *candida* PCR, and T2Candida panel) for IC and culture data from non-sterile sites (at least two extra-intestinal sites of *Candida* colonization) [33].

We found a 100% agreement among the experts. This suggests that echinocandins are favored as the first-line empiric therapy for IC in non-neutropenic ICU patients due to their efficacy and favorable safety profile [33]. Conversely, amphotericin B deoxycholate is avoided as empiric therapy due to its nephrotoxicity and high rates of infusion-related reactions [34,59]. Moreover, empiric AFT may be recommended for ICU patients with clinical evidence of intra-abdominal infection that is not responding to broad-spectrum antibiotics and who have predisposing factors for candidiasis, such as recent abdominal surgery or necrotizing pancreatitis [33]. The decision to discontinue empiric therapy should be considered if there is no clinical response within 4–5 days or no subsequent evidence for IC [33].

Neonatal IC poses a significant threat to the health and survival of premature infants. Challenges in accurately diagnosing this condition, coupled with the elevated risk of death or significant neurodevelopmental issues despite receiving proper treatment, underscore the critical importance of implementing effective preventative strategies [60]. There is 78% agreement where experts agree that empiric treatment for *Candida* infection might be advised for neonates in the ICU who are afflicted with conditions such as necrotizing enterocolitis, prematurity, and low birth weight [57].

In non-neutropenic patients, initiating empiric AFT depends on various clinical factors and diagnostic considerations, such as serum GM detection in serum or BAL. The detection of serum GM is less sensitive for diagnosing IA in non-neutropenic patients than in BAL, compared to neutropenic patients, as reflected by 100% agreement among experts [44]. When imaging studies reveal abnormalities suggestive of IA, but microbiological tests are inconclusive, a biopsy is recommended to confirm the diagnosis and exclude other infectious etiologies [44].

**Q2: Relevance of early empiric treatment approach in the changing IFD landscape (including epidemiology, between clinical guidelines and clinical practice).**

Initially, four statements addressed this question, and they were merged after voting into three statements, all of which reached a consensus.

The approach to AFT for IFDs is evolving alongside the changing IFD landscape. The early rationalization of empiric therapy is based on access to on-site, rapid, and timely fungal diagnostic modalities, including antigen testing and molecular strategies, leading to improved antifungal prescribing quality. While early initiation of antifungal treatment is crucial for high-risk hematology patients, given the considerable fatality rates caused by IFDs [43], concerns about antifungal resistance due to overuse necessitate a more pragmatic approach [56]. There is an ongoing debate on balancing the need for timely treatment with the potential downsides of broad-spectrum empiric AFT. The introduction of rapid and accurate fungal diagnostic tools, like antigen testing and molecular techniques, is paving the way for more targeted antifungal prescribing [61]. This indicates a potential shift from purely empirical approaches towards a more personalized treatment strategy based on confirmed fungal identification. Furthermore, we found 100% agreement among experts, suggesting the implementation of antifungal stewardship programs to optimize therapy rather than using empiric antifungals randomly based on best practice and clinical experience [56]. The latter suggests that complete abandonment of empiric treatment may be premature. Current clinical guidelines often acknowledge the pragmatic value of empiric therapy for high-risk patients, including those with neutropenia and persistent fever, particularly in settings with high local incidence of febrile neutropenia (IFN) and limited diagnostic capabilities, while advocating for careful consideration of the risk of overtreatment [43].

Therefore, the optimal approach is a judicious combination of both strategies. Early antifungal treatment should be based on a risk–benefit assessment, considering factors like the patient’s immune status, local IFD epidemiology, and the availability of rapid diagnostic tools. This will require ongoing collaboration among clinicians, microbiologists, and infectious disease specialists to ensure effective and responsible antifungal therapy.

**Q3: What are the clinical situations or patient profiles that require the early use of AFT?**

Three statements address this question, and all of them reached a consensus. Early AFT is crucial in specific clinical scenarios and patient profiles to improve outcomes. The present consensus document found 100% agreement among the experts, underscoring that immunocompromised adults, particularly hematological patients with a strong suspicion of invasive pulmonary aspergillosis (IPA), warrant prompt initiation of antifungal treatment alongside ongoing diagnostic evaluation [48]. This highlights the importance of early intervention, even before a definitive diagnosis, in patients at high risk of developing the condition. Similarly, we found 100% agreement among the experts, suggesting that early antifungal treatment is considered for pediatric hematological patients exhibiting any signs of IA, whether clinical, radiological, or microbiological [62]. This approach emphasizes the need for a low threshold for antifungal initiation in this vulnerable population. Furthermore, for immunocompromised adults with suspected mucormycosis, immediate initiation of AFT is paramount to maximize survival rates, upon which the agreement is 100% [63].

Presumptive tests, such as the KOH wet mount, GM assay, and molecular techniques, can be used to diagnose or routinely screen high-risk populations in a short timeframe, typically ranging from minutes to hours. This approach allows for the prompt initiation of empiric yet targeted therapy as soon as possible and improves survival rates [61]. For instance, the detection of characteristic hyphae provides a presumptive diagnosis of mucormycosis and the timely initiation of liposomal amphotericin B therapy. Additionally, PCR can differentiate between *Mucorales* species, enabling even more targeted antifungal treatment [64,65].

**Q4: Should additional factors (local epidemiology, antifungal prophylaxis) be considered when choosing an antifungal for empiric therapy to form antifungal stewardship programs?**

Two statements address this question, and both of them reached a consensus. As highlighted previously, patient and hospital-related factors are crucial [43,57]. The local epidemiology of IFDs is paramount. If a particular fungus predominates in a given hospital, tailoring empiric therapy to target that specific organism can lead to improved outcomes. Analyzing local data on IFDs helps to guide the initial choice of antifungal.

Beyond local epidemiology, patient-specific characteristics further refine the selection of empiric AFT, and 100% of the experts agree. A thorough evaluation of the patient’s medical history, current medications, and risk factors for IFD is essential. A patient’s prior AFT history and the prevalence of antifungal resistance within the hospital are equally important. Starting a patient on an antifungal when they have already failed, or using one with high local resistance, is unlikely to be effective [48]. Moreover, the type of primary antifungal drug used for prophylaxis in hematology patients may determine the choice of empiric antifungal agents, as reflected by 100% agreement on this statement (Table 1).

**Q5: What are the clinical situations or patients’ profiles that require a quick class switch? (Indications for switching between the antifungal classes).**

Initially, four statements addressed the question above, and they were divided into five after voting (statements 31 to 35), with percentages of agreement ranging from 78% to 100%. Several clinical scenarios necessitate a swift switch to a different antifungal class when treating IA and IM. All experts agree (100% agreement) that a switch is recommended for patients with refractory IA, where the initial antifungal fails to control the infection [66]. If a patient does not improve after a reasonable time (3–7 days) on an antifungal, it suggests treatment failure [48]. This could be due to fungal resistance or a misdiagnosis. For example, a worsening fever in an aspergillosis patient on azoles might necessitate switching to a different class [67]. Recognizing these signs early on enables clinicians to switch to an alternative antifungal class, thereby improving outcomes. This highlights the importance of monitoring treatment response and being prepared to adjust therapy if needed. For patients with breakthrough IA who were previously on triazole prophylaxis or treatment (except for fluconazole, as it has no activity against IA), switching to liposomal amphotericin B is often the next step [28,62,66]. This emphasizes the need for a switch to a different antifungal class to overcome potential intrinsic or emerging resistance during initial therapy. For patients with IM, combination therapy is desirable, especially among patients with refractory IM (Table 2). For both IA and IM, experiencing significant side effects from the current antifungal treatment justifies a switch to a different class [48]. For instance, switching from amphotericin B due to nephrotoxicity or away from azoles due to hepatoxicity would be a necessary course of action.

**Q6: What is the best time to switch from intravenous (IV) to per os (PO) antifungal therapy? What are the clinical signs and symptoms that may trigger a switch from IV to PO?**

There were six statements addressing this question, and all reached a consensus. Among them, statement 36 discusses the general switching principle, statements 37–38 consider IA, statements 39–40 refer to IC, and statement 41 focuses on invasive mucormycosis.

The decision to switch from IV to PO AFT depends on balancing treatment efficacy and patient comfort. Here is a breakdown of key considerations:

**General Principles:** Clinical and Microbiological Response: The switch can be considered when the patient shows satisfactory clinical improvement (reduced fever, improved inflammation markers) and/or a positive microbiological response (e.g., clearance of the fungus from blood cultures) [57,66]. This indicates a successful response to the initial IV therapy.

**Good Absorption and Drug Monitoring:** Switching to oral antifungals is only appropriate if the specific medication has good bioavailability (gut absorption) and therapeutic drug monitoring is available to ensure adequate blood levels [66].

**Specific Considerations by Disease:**

**Invasive Aspergillosis:** Voriconazole, posaconazole, and isavuconazole are all potential options for oral maintenance, depending on the availability of therapeutic drug monitoring to optimize dosing for each medication [66]. Notably, the switch should only be made in clinically stable patients with reliable gut absorption, as reflected by 100% of the agreement of the experts [66].

**Invasive Candidiasis:** For non-neutropenic patients with hemodynamic stability, who tolerate oral medications well, who have been afebrile for over 24 h, and who have cleared *Candida* from the bloodstream, switching from an echinocandin antifungal to oral fluconazole or voriconazole is possible [33,56,58,,68,69]. An early switch to oral azoles is recommended when the patient shows clinical improvement and blood cultures become negative [69]. Given the decreased susceptibility of some invasive *Candida* species to fluconazole, antifungal susceptibility testing for all *Candida* isolates from sterile sites should guide the management of invasive candidiasis [33].

**Invasive Mucormycosis:** Initial therapy for mucormycosis should consist of high-dose liposomal amphotericin B (5–10 mg/kg/day) administered intravenously [63,70,71,72,73]. In this case, IV treatment should continue until the disease stabilizes. All experts agree that isavuconazole or posaconazole are the preferred choices when transitioning to oral therapy due to their favorable absorption properties [70].

In conclusion, the switch from IV to oral AFT requires careful consideration of clinical response, the enteric absorption of the medication, and the availability of drug monitoring. By following these guidelines and tailoring the approach according to the specific fungal infection, clinicians can optimize the treatment efficacy while improving patients’ comfort and reducing healthcare costs.

**Q7: What are the benefits of early antifungal treatment in reducing mortality rate, hospital/ICU stay, and time to clinical improvement in different patient populations?**

There were six statements addressing this question, and all of them reached a consensus. Among them, statements 42–44 focus on hematology patients, and statements 45–47 explore ICU patients.

**a.**  **Hematology Patients:**

Early and aggressive management, including antifungal treatment, is crucial for mucormycosis, a fungal infection that often has poor outcomes if left untreated. This approach improves survival rates, especially in patients with hematological malignancies and immunocompromised children [63,70,71,72,73]. In neutropenic patients with persistent fever, which can be a sign of invasive fungal disease, early empiric AFT can reduce the incidence and severity of the underlying infection [74,75]. Notably, studies show that voriconazole use is associated with higher survival rates in hematology patients suffering from acute respiratory failure and IPA [76].

**b.**  **ICU Patients:**

Identifying critically ill patients at high risk for IFDs is paramount for the successful implementation of early antifungal strategies. While general risk factors apply, certain ICU populations carry a significantly elevated risk for specific fungal pathogens. These high-risk groups, including patients affected by the recent pandemic, are detailed in Table 3. For example, the global COVID-19 pandemic significantly altered the landscape of IFDs in the ICU, leading to a dramatic increase in patients susceptible to both COVID-19-associated pulmonary aspergillosis (CAPA) and severe candidemia. This susceptibility is largely attributed to severe viral-induced lung damage, prolonged mechanical ventilation, and the use of high-dose corticosteroids or immunomodulators like tocilizumab in this population [77,78].

Studies suggest that early empiric AFT in high-risk ICU patients can lead to a lower incidence of IC, potentially reducing hospital resource utilization [56,79]. Conversely, delayed treatment of IA in critically ill patients is associated with a high mortality rate, highlighting the importance of timely intervention [61]. Early AFT in this population may contribute to decreased morbidity and mortality [43]. Table 4 summarizes Q7 statements.

**Table 4:** Experts’s tatements and recommendations on benefits of early antifungal treatment.

Number	Statement/Recommendation	Percentage of Agreement	Level of Evidence
**Q7. Benefits of early antifungal treatment** **in reducing mortality rate** **, hospital/ICU stay, and time to clinical improvement in different patient populations.**			
**Hematology patients:**			
**42** **.**	In the case of mucormycosis, the application of early, multidisciplinary treatment approaches, including aggressive surgical debridement, improves the survival rate, particularly in patients with hematological malignancies.	*100%*	IV
**43.**	The use of empiric antifungal therapy in neutropenic patients with persistent fever reduces the incidence and morbidity of invasive fungal diseases	*100%*	I
**44.**	Voriconazole use is associated with higher survival rates of hematology patients suffering from acute respiratory failure and invasive pulmonary aspergillosis	*78%*	IV
**ICU patients:**			
**45.**	Early empiric treatment in high-risk populations is associated with a low incidence of invasive candidiasis in critically ill patients	*78%*	IV
**46.**	Invasive aspergillosis is associated with a high mortality rate in critically ill patients if not adequately treated	*100%*	V
**47.**	Early antifungal treatment may be used to decrease the morbidity and mortality of critically ill patients and immunocompromised patients	*89%*	V

Abbreviations: ICU, intensive care unit.

The limitations of this consensus document include the small sample size of the involved HCPs and the risk of biased judgment of those experts based on their local experience and clinical practice, leading to potential selection, response, or confirmation bias.

This consensus recommends empiric AFT for high-risk patients, including febrile neutropenic hematology patients on long-term antibiotic use and critically ill non-neutropenic patients with unexplained fever and risk factors for fungal infections. It emphasizes the importance of considering local epidemiology and antifungal stewardship programs when implementing this approach. Additionally, the consensus highlights the importance of early initiation of AFT for reducing mortality and hospital stay and improving response time in immunocompromised patients with suspected IFDs.

Furthermore, the implementation of environmental preventive measures is essential in managing high-risk patients. These measures include performing an infection risk assessment, maintaining air quality through the use of HEPA filtration, ensuring direct room airflow, maintaining positive room air pressure, and ensuring well-sealed rooms. Additionally, these measures include continuous surveillance, antifungal prophylaxis, and patient and staff education [80,81].

Preventive measures to minimize airborne fungal spores during construction and renovation involve site containment, installing barriers to separate construction zones from clinical areas, regularly cleaning the construction site, employing negative air-pressure systems with exhaust fans, and deploying portable HEPA filters at the site [82].

## 4. Conclusions

Early and targeted antifungal treatment is critical for high-risk patients with invasive fungal infections. Physicians should initiate empiric therapy with the most appropriate antifungal class before the definitive diagnosis, taking into account factors such as local fungal prevalence and the patient’s medical history. Close monitoring is essential for timely switching to a different class of antifungal medication to reduce mortality, especially if the patient does not improve within expected time limits or experiences severe side effects. Ultimately, the successful transition from intravenous to oral medication depends on the specific infection and the patient’s progress. By implementing this targeted approach and starting treatment early, clinicians can significantly improve outcomes for these vulnerable patients.

## Figures and Tables

**Table 1:** Experts’ statements and recommendations on when to consider early empiric treatment.

Number	Statement/Recommendation	Percentage of Agreement	Level of Evidence
**Q1. When is the empiric approach appropriate and should be implemented (what types of centers, diagnostic tools, and clinical situations)?**			
**Hematology patients:**			
**1.**	It is recommended to investigate the presence of invasive fungal disease using appropriate diagnostic approaches before initiating empiric antifungal therapy	*100%*	V
**2.**	The recommended diagnostic approach for neutropenic patients with suspicion of invasive aspergillosis (IA) is a chest computed tomography (CT) scan, serum and/or bronchoalveolar lavage (BAL) galactomannan antigen (GM) detection	*100%*	V
**3.**	Whenever possible, the microscopy examination with fluorescent brighteners that are used as enhancing methods for the detection of fungal elements, such as Calcofluor White (CFW) and Blankophor, in BAL specimens and histopathologic examination of tissues and fluid specimens’ cultures are recommended in case of suspected IA	*100%*	IV
**4.**	Empiric therapy may be recommended in those settings where diagnostic abilities are not easily available	*89%*	V
**5.**	If empiric therapy is indicated in hematology patients, liposomal amphotericin B, caspofungin, micafungin, or isavuconazole may be used. Voriconazole is recommended in cases of suspected invasive aspergillosis	*78%*	V
**6.**	Empiric antifungal therapy is not recommended for patients who are anticipated to have short durations of neutropenia (less than 10 days) unless other findings indicate a suspected invasive fungal infection	*89%*	V
**7.**	In patients with suspected breakthrough invasive fungal infection, antifungal therapy should be initiated using an antifungal agent of a different class until the diagnosis is confirmed and response to treatment can be documented	*100%*	V
**8.**	The diagnostic approaches for pediatric patients are the same as those for adults	*89%*	V
**9.**	Empiric antifungal therapy (if chosen as a strategy) may be recommended for children with acute myeloid leukemia, high-risk lymphoblastic leukemia or relapsed acute leukemia with prolonged neutropenia; on high-dose steroids and underwent allogeneic hematopoietic stem cell transplantation (HSCT) after four to seven days of fever with unclear reason and unresponsive to broad-spectrum antibacterial agents.	*100%*	V
**10.**	Caspofungin (50 mg/m^2^ per day; day 1, 70 mg/m^2^; maximum 70 mg per day) and liposomal amphotericin B (3 mg/kg per day) are recommended as empiric treatment for pediatric patients	*89%*	II
**11.**	Empiric antifungal therapy should be continued in children with granulocytopenia until recovery from neutropenia, even in the absence of suspected or documented invasive fungal disease	*88%*	V
**Non-neutropenic ICU patients:**			
**12.**	Bronchoscopy with fungal cultures and GM in BAL are recommended diagnostic tools in critically ill patients with suspected IA	*100%*	V
**13.**	Serum GM is not routinely recommended in case of non-neutropenic critically ill patients with suspicion of IA	*78%*	IV
**14.**	Incorporating the conventional culture-based tests and the non-culture-based tests (serological tests, miniaturized-magnetic resonance-based technology, and PCR-based tests) is recommended as part of the diagnostic approach for IC	*89%*	V
**15.**	Antifungal therapy may be considered in critically ill patients with one or more of the following characteristics: multi-organ failure, septic shock, prolonged ICU stay, high Acute Physiology and Chronic Health Evaluation II (APACHE II) scores, multifocal *candida* colonization, and no identifiable cause of fever. It should be based on a clinical assessment of risk factors, supported by one or more surrogate markers (beta-D-glucan, *Candida* PCR, and T2Candida panel) for invasive candidiasis, and culture data from non-sterile sites (at least two extra-intestinal sites of *Candida* colonization).	*89%*	V
**16.**	Echinocandin is considered the preferred antifungal for empiric therapy for invasive candidiasis in non-neutropenic patients in the ICU	*100%*	V
**17.**	Amphotericin B deoxycholate is not recommended as empiric therapy because of its nephrotoxicity and high rates of infusion-related reactions	*89%*	IV
**18.**	Antifungal therapy in ICU patients with catheter-related candidemia should be started, along with catheter removal	*100%*	V
**19.**	Empiric antifungal therapy for candidiasis may be recommended for ICU patients with intra-abdominal infection not responding to the broad-spectrum antibiotics	*78%*	V
**20.**	Empiric antifungal therapy for candidiasis may be recommended in cases of necrotizing enterocolitis, prematurity, and low birth weight for the neonatal ICU	*78%*	V
**21.**	GM detection in serum is less sensitive in non-neutropenic patients for diagnosis of IA than in bronchoalveolar lavage, compared with neutropenic patients	*100%*	V
**22.**	If an abnormality is detected on a CT scan and the microbiological tests are negative, it is suggested to perform a biopsy to confirm IA and exclude other infections	*89%*	V
**Q2. Relevance of early empiric treatment approach in the changing IFD landscape (including epidemiology, between clinical guidelines and clinical practice).**			
**23.**	The early rationalization of empiric therapy is based on access to on-site, rapid, and timely fungal diagnostic modalities, including antigen testing and molecular strategies, leading to improved antifungal prescribing quality	*100%*	V
**24.**	It is recommended to promote antifungal stewardship programs to optimize the use of antifungal therapy. (best practice statement)	*100%*	V
**25.**	In those settings with a high incidence of IFD and with limited diagnostic facilities, empiric antifungal therapy is a reasonable and pragmatic alternative in hematology patients with neutropenia and persistent fever.	*89%*	V
**Q3. What are the clinical situations or patient profiles that require the early use of AFT?**			
**26.**	It is recommended to initiate the antifungal treatment early in adult patients with hematological malignancies and with strongly suspected invasive pulmonary aspergillosis (IPA) while a diagnostic evaluation is conducted	*100%*	V
**27.**	Early antifungal treatment is considered in pediatric hematological patients with at least one positive clinical, imaging, or microbiologic feature suggesting invasive aspergillosis	*100%*	V
**28.**	Immediate initiation of treatment in immunocompromised adult patients with suspected mucormycosis is recommended to increase the survival rate	*100%*	V
**Q4. Should additional factors (local epidemiology, antifungal prophylaxis) be considered when choosing an antifungal for empiric therapy in the formation of antifungal stewardship programs?**			
**29.**	Empiric strategies should be selected based on patient- or hospital-related factors. These include accessibility to diagnostic tools and drugs, feasibility, local epidemiology, prior AFT, prevalence of antifungal resistance, risk of IFD, adverse effects, and pharmacoeconomic issues	*100%*	V
**30.**	The choice of empiric antifungal agents may be based on the type of antifungal drug used for primary prophylaxis in hematology patients	*100%*	V

Abbreviations: AFT, antifungal therapy, APACHE II, Acute Physiology and Chronic Health Evaluation II; BAL, bronchoalveolar lavage; CFW, calcofluor white; CT, computed tomography; GM, galactomannan antigen; HSCT, hematopoietic stem-cell transplant; IA, invasive aspergillosis; IC, Invasive candidiasis, ICU, intensive care unit; IFD, invasive fungal diseases; IPA, invasive pulmonary aspergillosis; PCR, polymerase chain reaction.

**Table 2:** Experts’ statements and recommendations on quick class switches and timing regarding early antifungal treatments.

Number	Statement/Recommendation	Percentage of Agreement	Level of Evidence
**Q5. What are the clinical situations or patient profiles that require a quick class switch? (Indications for switching between the antifungal classes)**			
**Invasive Aspergillosis**			
**31** **.**	In case of refractory invasive aspergillosis, switching to antifungal agents from a different class is recommended	*100%*	V
**32.**	Switching to liposomal amphotericin B is considered in case of breakthrough invasive aspergillosis occurring on triazole (except for fluconazole, as it has no activity against IA) prophylaxis or treatment	*100%*	III
**33.**	For the management of invasive aspergillosis, switching to a different antifungal class is recommended in patients who experience an adverse event attributable to the current antifungal agent	*89%*	V
**Invasive Mucormycosis**			
**34.**	In case of refractory mucormycosis, combination therapy may be considered.	*89%*	V
**35.**	In case of toxicity of first-line regimens (nephrotoxicity and hepatoxicity, which are related to amphotericin B), switching to azole drugs (such as isavuconazole and posaconazole) is recommended	*89%*	V
**Q6. What is the best time to switch from intravenous (IV) to per os (PO) antifungal therapy? What are the clinical signs and symptoms that may trigger a switch from IV to PO?**			
**36.**	Switching from IV to PO antifungal therapy should be considered in centers where drug monitoring is available and in patients who show satisfactory clinical and/or microbiological response, have good enteric absorption	*100%*	V
**Invasive Aspergillosis**			
**37.**	Voriconazole, posaconazole, and isavuconazole can be used interchangeably based on the availability of therapeutic drug monitoring	*78%*	V
**38.**	Switching from intravenous to oral therapy should be recommended for patients who are clinically stable and have a reliable enteric absorption	*100%*	V
**Invasive Candidiasis**			
**39.**	Switching from echinocandin to oral fluconazole or voriconazole is considered in non-neutropenic patients with hemodynamic stability, who are afebrile for more than 24 h, can tolerate oral therapy, and have *Candida* cleared from the bloodstream, and when the isolate is confirmed susceptible to the chosen azole agent	*78%*	V
**40.**	It is recommended to step down to oral azole as early as possible once the patient is clinically stable and blood cultures have become negative	*89%*	V
**Invasive Mucormycosis**			
**41.**	Intravenous treatment with high-dose (5–10 mg/kg/day) liposomal amphotericin B is recommended until the disease is stable. When switching to oral therapy, the use of isavuconazole or posaconazole is recommended	*100%*	V

Abbreviations: IV, intravenous; PO, per os.

**Table 3:** High-Risk ICU Patient Populations for Invasive Fungal Diseases.

Risk Category	Clinical Characteristics	Associated IFDs
**COVID-19-associated**	Severe COVID-19 requiring ICU admission, prolonged mechanical ventilation, corticosteroid therapy, tocilizumab use	COVID-19-associated pulmonary aspergillosis (CAPA), candidemia
**Septic shock/Multi-organ failure**	APACHE II score >20, vasopressor requirement, multiple organ dysfunction	Invasive candidiasis, breakthrough aspergillosis
**Post-surgical/Trauma**	Abdominal surgery, necrotizing pancreatitis, major trauma with prolonged ICU stay	Intra-abdominal candidiasis, surgical site infections
**Immunosuppressed ICU patients**	Solid organ transplant recipients, chronic corticosteroids, immunomodulatory therapy	Invasive aspergillosis, mucormycosis, candidiasis

Abbreviations: IFD, Invasive Fungal Disease; ICU, Intensive Care Unit; CAPA, COVID-19-associated Pulmonary Aspergillosis; APACHE II, Acute Physiology and Chronic Health Evaluation II.

## Data Availability

No new data were created or analyzed in this study. Data sharing is not applicable to this article.

## Abbreviations

The following abbreviations are used in this manuscript:

AFT	Antifungal Therapy
AML	Acute Myeloid Leukemia
APACHE	Acute Physiology and Chronic Health Evaluation II
AST-IDCOP	American Society of Transplantation Infectious Diseases Community of Practice
BAL	Bronchoalveolar Lavage
BDG	β-(1,3)-d-glucan
CAGTA	*C. albicans* Germ Tube Antibody
cfDNA	Cell-Free DNA
CFW	Calcofluor White Stain
CT	Computed Tomography
EAT	Empiric Antimicrobial Therapy
ECIL	The European Conference on Infections in Leukemia
GM	Galactomannan Antigen
HCPs	Healthcare Professionals
HSCT	Hematopoietic Stem Cell Transplant
IA	Invasive Aspergillosis
IC	Invasive Candidiasis
ICU	Intensive Care Unit
IDSA	Infectious Diseases Society of America
IFDs	Invasive Fungal Diseases
IM	Invasive Mucormycosis
IPA	Invasive Pulmonary Aspergillosis
IV	Intravenous
PCR	Polymerase Chain Reaction
PO	Per Os

## Appendix A

**Table A1:** Levels of evidence and grades of agreement.

Levels of Evidence	
**I**	Evidence from at least one large randomized, controlled trial of good methodological quality (low potential for bias) or meta-analyses of well-conducted randomized trials without heterogeneity
**II**	Small randomized trials or large randomized trials with a suspicion of bias (lower methodological quality) or meta-analyses of such trials or trials demonstrating heterogeneity
**III**	Prospective cohort studies
**IV**	Retrospective cohort studies or case–control studies
**V**	Studies without a control group, case reports, or expert opinions
